# 通用型嵌合抗原受体T细胞治疗的研究进展

**DOI:** 10.3760/cma.j.issn.0253-2727.2021.09.015

**Published:** 2021-09

**Authors:** 玥 黄, 珂嘉 胡, 永仙 胡, 河 黄

**Affiliations:** 浙江大学医学院附属第一医院，杭州 310003 The First Affiliated Hospital, School of Medicine, Zhejiang University, Hangzhou 310003, China

嵌合抗原受体T细胞（CAR-T细胞）疗法是一种新型免疫细胞治疗，CAR主要由识别肿瘤特异性抗原的单链抗体（scFV）段、铰链区及跨膜段、共刺激分子结构域、CD3ζ结构域构成，通过将编码CAR的基因引入T细胞，使T细胞实现不依赖于主要组织相容性复合体（MHC）抗原递呈系统的特异性杀伤。CAR-T细胞近年来在治疗血液系统恶性肿瘤方面取得了巨大突破，显著提高了各种血液肿瘤患者的完全缓解率及长期生存率。最新临床研究显示，CAR-T细胞治疗难治复发急性B细胞白血病、B细胞淋巴瘤和多发性骨髓瘤完全缓解率分别达81％[1]、52％～67％[2]、73％～85％[3]。

目前上市的CAR-T细胞均为自体细胞来源，自体来源的CAR-T细胞存在以下缺陷：①婴幼儿或经过多线治疗的患者难以采集足够数量的外周血T细胞或T细胞功能受损导致其制备的产品质量下降；②由于自体CAR-T细胞不能大规模生产，使用次数及剂量受患者自身情况所限；③自体CAR-T细胞的生产成本较高；④自体CAR-T细胞从采集到回输需要一定的时间，有可能导致患者错过最佳治疗时机；⑤CAR-T产品中易混入患者自身的肿瘤细胞。为此，自体CAR-T细胞的应用受到了限制，而通用型CAR-T细胞将成为这一领域的重要发展方向。2017年至2021年，全球29项通用型CAR-T细胞治疗临床试验已开始进行，临床试验数目逐年扩增（表1）。但尽管如此，通用型CAR-T细胞仍然面临重大挑战。本文从免疫排斥、细胞持久性和底盘细胞来源三个角度对目前通用型CAR-T细胞治疗中存在的问题和应对策略进行总结。

一、通用型CAR-T细胞治疗的免疫排斥

通用型CAR-T细胞治疗中最大的挑战在于宿主细胞对通用型CAR-T细胞的免疫排斥以及通用型CAR-T细胞攻击宿主组织器官。αβT细胞占T细胞总数95％以上，αβT细胞的T细胞受体（TCR）由α链（TRAC基因编码）、β1链（TRBC1基因编码）和β2链（TRBC2基因编码）构成，同种异基因CAR-T细胞表面TCR可以识别宿主细胞抗原，引发移植物抗宿主病（GVHD）。此外，同种异基因CAR-T细胞表面的MHC，又称为人类白细胞抗原（HLA），诱发宿主TCR识别，引起免疫排斥。HLA可分为HLA Ⅰ类分子和HLA Ⅱ类分子，前者由具有高度多态性的α链和由B2M基因编码的β链（即β_2_微球蛋白）构成；后者被发现高表达于活化的T细胞表面[4]，为介导受体CD4+ T细胞发生免疫排斥的关键分子[2]。

得益于锌指核糖核酸酶（ZFN）、转录激活因子样效应因子核酸酶（TALEN）、CRISPR/Cas9等多种基因编辑技术的发展，我们能够对CAR-T细胞TCR和HLA进行敲除，以实现异基因T细胞免疫原性的消除，降低免疫排斥和GVHD。2012年，美国Anderson癌症中心的Torikai团队首次报道利用ZFN技术敲除CAR-T细胞表面TCRα或β链。CAR^+^TCR^−^ T细胞不响应TCR刺激，且展现出良好的抗B细胞肿瘤治疗的有效性和安全性，成为通用型CAR-T的最初模型[6]。使用相同技术进一步敲除CAR-T细胞表面HLA-A位点，构建HLA-A^−^ CAR-T细胞，能够消除宿主的免疫排斥反应[7]。此外，敲除编码HLA Ⅱ类分子α链的基因（HLA-DRA、DQA和DPA基因）[8]，也显示出良好的降低免疫原性作用。

CRISPR/Cas9技术相比于ZFN和TALEN，有更高的基因位点选择性和准确性。将CAR载体定点插入基因组中可增加CAR表达的均一性，2017年Sadelain团队首次报道将CAR插入TRAC基因座中，可减少CAR信号通路的持续激活以延缓CAR-T细胞的分化和衰竭，增加CAR-T细胞的抗肿瘤活性[9]。同年，宾夕法尼亚大学Ren等[10]运用CRISPR/Cas9技术实现CAR-T细胞TCR和B2M双敲除，输注后未发生GVHD；在此基础上设计的TCR、B2M和PD1三敲除CAR-T细胞进一步显示出更显著的T细胞抗肿瘤活性。2019年耶鲁大学陈斯迪团队开发AAV-Cpf1 KIKO技术，建立了一个稳定的双CAR敲入和免疫检查点敲除系统，一步法制备CD22BBz和CD19BBz双敲入的同时破坏TRAC及PDCD1功能的工程改造T细胞，在体内外均表现出强效的细胞因子分泌功能和细胞毒性[11]。

在临床应用方面，法国Cellectis公司联合伦敦大学设计由TALEN技术敲除TCR α/β链和CD52的抗CD19 UCART细胞（UCART19）[12]。TCR α/β链的缺乏消除了GVHD反应，而CD52的敲除使得CAR-T细胞获得对alemtuzumab（阿仑单抗，用于消除宿主T细胞的抗CD52单克隆抗体）的耐受性。随后1例骨髓移植后复发的急性淋巴细胞白血病（ALL）儿童患者接受上述UCART治疗，成功达到分子生物学缓解，无GVHD反应[13]，实现了通用型CAR-T细胞治疗在临床应用的里程碑式突破。在此基础上，一系列通用型CAR-T细胞临床试验陆续开展（表1）。

**表1:** 通用型CAR-T细胞治疗临床试验^a^（2017—2021）

注册号	开始招募时间	试验阶段	靶点	研究机构	疾病
NCT03166878	2017/6/1	1期/2期	CD19	解放军总医院	B细胞白血病、B细胞淋巴瘤
NCT03190278	2017/6/19	1期	CD123	法国Cellectis公司	难治复发急性髓系白血病
NCT03389035	2017/12/20	1期/2期	CD19	Matilde Tettamanti Menotti De Marchi Onlus基金会	复发后急性淋巴细胞白血病
NCT03398967	2018/1/2	1期/2期	CD19+CD20/CD22（双靶）	解放军总医院	B细胞白血病、B细胞淋巴瘤
NCT03229876	2019/6/1	NA	CD19	郑州大学第一附属医院、浙江大学附属第一医院	急性淋巴细胞白血病、非霍奇金淋巴瘤
NCT04173988	2020/1/9	1期（早）	CD19	上海复旦大学附属儿童医院	儿童急性淋巴细胞白血病
NCT03666000	2019/3/11	1期/2期	CD19	美国希望之城医疗中心等	急性B淋巴细胞白血病、非霍奇金淋巴瘤
NCT04150497	2019/10/14	1期	CD22	法国Cellectis公司	急性B淋巴细胞白血病
NCT03752541	2019/11/1	NA	BCMA	上海邦耀生物科技有限公司、同济大学医学院附属上海同济医院、中南大学湘雅第二医院	多发性骨髓瘤
NCT04142619	2019/11/21	1期	CS1	法国Cellectis公司	难治复发骨髓瘤
NCT04227015	2020/1/8	1期（早）	CD19	浙江大学附属第一医院	急性淋巴细胞白血病、非霍奇金淋巴瘤
NCT04173988	2020/1/9	1期（早）	CD19	复旦大学附属儿童医院	儿童急性B淋巴细胞白血病
NCT04230265	2020/1/28	1期	CD123	日本Cellex公司	急性髓系白血病、急性B淋巴细胞白血病、母细胞性浆细胞样树突状细胞肿瘤
NCT04384393	2020/5/9	1期	CD19	中国科学技术大学附属第一医院（安徽省立医院）	B细胞肿瘤
NCT04557436	2020/8/12	1期	CD19	英国伦敦大学	急性B淋巴细胞白血病
NCT04538599	2020/9/1	NA	CD7	浙江大学附属第一医院	T细胞恶性血液肿瘤
NCT04556266	2020/9/14	1期	CD19	美国西雅图儿童医院	B细胞白血病、B细胞淋巴瘤
NCT04288726	2020/9/16	1期	CD30	美国贝勒医学院	结外型NK/T细胞淋巴瘤、鼻型/经典型霍奇金淋巴瘤
NCT04601181	2020/10/23	1期	CD22	中国科学技术大学附属第一医院（安徽省立医院）	B细胞恶性肿瘤
NCT04613557	2020/11/26	1期	BCMA	比利时Celyad Oncology公司	难治复发多发性骨髓瘤
NCT04176913	2020/12/1	1期	CD20	江苏省人民医院	B细胞淋巴瘤
NCT04796688	2021/3/10	1期	CD19	华中科技大学同济医学院附属协和医院、成都美斯诺生物科技有限公司	急性淋巴细胞白血病、慢性淋巴细胞白血病、B细胞淋巴瘤
NCT04881240	2021/5/1	1期	CD19	美国St. Jude儿童研究医院	难治复发急性淋巴细胞白血病、儿童急性淋巴细胞白血病

注：NA：不适用；BCMA：B细胞成熟抗原。^a^所有临床试验目前状态均为招募中

除编辑CAR-T外，在宿主体内构建适合的免疫细胞环境也是降低免疫排斥的重要策略，临床上通常采用预处理化疗方案以构建患者体内低淋巴细胞环境。目前CAR-T细胞治疗前常用预处理方案为氟达拉滨+环磷酰胺（FC），而对于通用型CAR-T细胞，在结合基因编辑技术的基础上，需要更合适的淋巴细胞清除方案。早在2015年即有研究报道利用基因编辑技术敲除CAR-T细胞CD52基因，使得CAR-T细胞（UCART19）具有对阿仑单抗的抗性，同时在患者体内实现较为彻底的淋巴细胞清除预处理[14]。本研究团队应用抗CD19/CD22双靶点CAR-T产品CTA101（CRISPR基因编辑技术敲除TRAC位点以及CD52基因；并在此基础上使用慢病毒转染CAR基因及“自杀开关”RQR8），开展通用型CAR-T细胞治疗成人R/R ALL的1期临床试验（NCT04227015），联合阿伦单抗的通用型细胞治疗策略完全缓解率达83.3％（5/6），且无GVHD及死亡病例[15]。

在通用型CAR-T细胞治疗过程中，尽管通过基因编辑技术敲除B2M基因可降低HLA Ⅰ介导的免疫原性，然而HLA-I是NK细胞主要的抑制性受体之一，敲除TCR及HLA的CAR-T细胞仍无法逃避宿主NK细胞介导的免疫排斥[16]。针对NK细胞介导的排斥反应问题，国际上目前手段聚焦于抑制NK细胞的功能。Gornalusse等[17]在多能干细胞（PSC）的B2M基因座处敲入HLA-E基因，使得这些PSC表达HLA-E单链二聚体或三聚体，不被宿主CD8^+^ T细胞识别，同时对NK介导的细胞毒作用具有抗性。此外，过表达NK细胞的siglec 7和siglec 9配体能够介导NK细胞功能下调，也被认为是有效的方法[18]。但这类策略导致广泛抑制NK细胞作用被广泛抑制，致患者发生免疫抑制并出现感染等相关并发症。因此，如何特异清除异基因反应性的活化NK细胞，仍是亟待解决的问题。

二、通用型CAR-T细胞的持久性

多项试验表明，CAR-T细胞的疗效和预后与细胞在体内的持久性有密切关联。相比自体CAR-T细胞，异基因CAR-T细胞在体内的持久性明显降低。近期一项利用HLA全相合或半相合同种异基因CAR-T细胞治疗ALL的研究中，所有（8/8）患者的CAR-T细胞在输注后30 d内低于检测下限，其中4例HLA半相合CAR-T细胞治疗患者均在短期内复发死亡[19]。CAR-T细胞体内的分化能力是影响CAR-T细胞在体内持久性的重要因素。中央记忆型T细胞（Tcm）和干细胞样记忆型T细胞（Tscm）是两种能够在体内持久扩增细胞类型，提高输注前CAR-T产品中CAR-Tcm、CAR-Tscm的占比有利于细胞在体内的有效扩增。一方面，体外培养体系中的细胞因子成分被认为是影响CAR-T细胞亚群占比和细胞功能的重要因素，IL-7、IL-15和IL-21与Tm细胞的状态维持有关。IL-7和IL-21促进T细胞脂肪酸氧化，提高记忆性CD8^+^ T细胞的比例[20]–[21]；在IL-15条件下培养的CAR-T细胞（CAR-T/ILT15）表现出低分化Tscm表型，这与mTORC1活性下降和糖酵解酶表达减少密切相关[22]。另一方面，采用合适的CAR-T细胞共刺激域也是有效的策略，自体CAR-T细胞治疗临床试验数据表明，装载CD28共刺激域的CAR-T细胞在输注后68 d内CAR-T细胞完全耗竭[23]；相比之下，4-1BB/CD3-z CAR-T细胞产品tisagenlecleucel（Kymriah）有更明显的记忆样T细胞表型，tisagenlecleucel在外周血中位持续时间为168 d[24]，在中国人群体中4-1BB/CD3-z CAR-T细胞的最长存活时间为7个月[25]。

此外，已有多篇文献报道在T细胞里过表达记忆细胞形成相关的转录因子，如c-JUN[26]和c-Myb[27]等，可以提高T细胞的体内持久性。得益于基因编辑技术的发展，通用型CAR-T能够在此基础上对细胞耗竭、细胞记忆性相关转录因子进行编辑，提升CAR-T细胞产品的体内持久性。本研究团队采用CRISPR/Cas9基因编辑技术对T细胞PDCD1基因进行特异性切割并导入CD19-CAR元件，该新型CAR-T细胞显著提高复发难治淋巴瘤患者中的疗效，不良反应轻且可控。

关于敲除TCR对CAR-T细胞在体内扩增的影响存在争议。有研究表明，CAR和TCR的双重激活可能是CD8^+^ CAR-T细胞凋亡增加及出现耗竭表型的一大原因，TCR的存在可能加快了CAR-T细胞在体内的耗竭[28]。因此，敲除内源性TCR可能有利于CAR-T细胞在体内的扩增。然而，也有实验表明内源性TCR的存在使得CAR-T细胞的扩增更为长久，在异种移植模型小鼠中，杀伤肿瘤的能力更强，被输注TCR^+^CAR-T细胞的小鼠的生存周期更长[29]。由于两者所用小鼠模型存在差异，CAR-T细胞上TCR的敲除对其扩增的影响尚待进一步研究。

三、底盘细胞的来源

如前文所述，αβT细胞所介导的免疫识别是引起GVHD的主要因素，研究者们尝试使用其他能够实现表面受体特异性重定向、同时易于体外扩增和转导的非αβT细胞。目前已有的研究包括NK细胞、巨噬细胞、γδT细胞和NKT细胞，在异基因细胞治疗中具有巨大的潜力[30]。

NK细胞应用最为广泛。目前体外研究的CAR-NK细胞主要来源于细胞系（主要为NK-92细胞系）、脐带血和外周血。相比于T细胞，NK细胞在接触抗原后无需经过分化即可激活产生免疫应答，且表面既表达识别MHC Ⅰ类分子的活化及抑制受体，也表达识别非MHC Ⅰ类分子的杀伤活化受体，因此在抗肿瘤免疫反应中发挥重要作用[31]。CAR-NK细胞相比CAR-T细胞在循环内存在的时间较短，因此产生的毒副作用低，显示出更好的安全性[32]。但临床试验显示，其体外扩增和体内持久性仍有待提高。目前已注册的CAR-NK临床试验除包括治疗血液肿瘤（NCT02944162、NCT02892695、NCT02742727、NCT03056339、NCT04796688、NCT04796675、NCT04639739、NCT04747093）和实体肿瘤（NCT02839954）。

属于固有免疫系统的γδT细胞表现出高效的细胞毒性且不受MHC限制而识别目标抗原[33]，且除TCR外，其表面还表达NK型受体如NKG2D，因此CAR-γδT细胞也可像CAR-NK细胞一样，允许除CAR引导的抗原识别反应外靶向其他多个肿瘤相关抗原[34]。目前已有多项临床试验开展，在治疗急性髓系白血病（NCT03790072）、B系淋巴系统疾病（NCT02656147）和急性T淋巴细胞白血病（NCT04702841）领域均展现出杰出的应用前景。

此外，NKT细胞和巨噬细胞也是底盘细胞的选择之一，NKT细胞主要识别鞘糖脂、糖脂或脂质抗原等，目前已有两项已开展的临床研究（NCT03294954和NCT03774654）。巨噬细胞作为重要的固有免疫细胞，M1型具有抗肿瘤免疫作用，如何维持CAR-巨噬细胞维持M1表型提高其抗肿瘤作用并减少全身毒性是其未来发展方向。

以上所提及的固有免疫细胞作为CAR的底盘细胞仍面临挑战，其中最主要的是细胞来源和感染效率问题。不同于T细胞，大部分固有免疫细胞在外周血白细胞中的比例并不高，NK细胞占2％～5％，γδT细胞占1％～5％，NKT细胞仅占0.01％～0.5％。巨噬细胞为单核细胞穿出血管后分化而成，几乎不可能从外周血中获得。因此不少研究针对从诱导干细胞分化而来，尤其是NK细胞和巨噬细胞。目前有部分研究聚焦于造血干细胞或诱导干细胞（iPSC），以作为通用型CAR底盘细胞的来源。目前已有团队成功制造了iPSC来源的CAR-NK细胞[35]和CAR-巨噬细胞[36]，并证明了其强大的抗肿瘤功能。其次，尽管固有免疫细胞难以被病毒感染，但随着非病毒转染细胞技术迅猛发展，一定程度上使得装载CAR的固有免疫细胞产品化成为可能。CAR-T细胞、CAR-NK细胞及CAR-γδT细胞优缺点见表2。

**表2:** 转导嵌合抗原受体（CAR）载体的不同底盘细胞优缺点比较

类型	优势	劣势
CAR-T	①在外周血中比例较高，易制备 ②易被病毒转染 ③体外扩增能力强 ④体内杀伤维持能力相对久	①识别同种异基因MHC分子，引起GVHD ②细胞表面存在免疫抑制点（如PD1、LAG3等），与相应配体结合后抑制抗肿瘤活性
CAR-NK	①不引起GVHD ②毒副作用小 ③有独立于CAR识别的非特异性杀伤肿瘤作用	①单次输注持久性有限 ②不易被病毒转染
CAR-γδT	①不引起GVHD ②毒副作用较小 ③有独立于CAR识别的非特异性杀伤肿瘤作用	①在外周血中比例极低，不易制备 ②单次输注持久性有限 ③不易被转染/转导

注：MHC：主要组织相容性抗原复合体；GVHD：移植物抗宿主病

四、总结与展望

全球正步入免疫治疗的新时代，CAR-T细胞治疗谱写了血液系统恶性肿瘤的治疗新前景。在此基础上开发“现货型”通用CAR-T细胞，能够以更低的成本、更模式化的生产工艺和更广泛的适应证显著增加此类疗法的应用。基因编辑技术的发展能够通过有效消除TCR、HLA的表达来控制GVHD风险的策略。尽管如此，在提高CAR-T细胞的功效以及宿主的免疫排斥方面，仍然存在许多挑战。但正如我们所讨论的，目前越来越多的技术和方法可以实现免疫治疗的优化。

我们认为，在已有的技术基础上，一方面，利用合成生物学方法设计可时空性调控细胞存续的通用型CAR-T，能够完善对靶抗原的筛选设计和对治疗安全性的优化，实现新型通用型CAR-T产品的临床设计；另一方面，基于原位单细胞检测技术的临床前及临床研究是下一代细胞免疫治疗的重要基石，能够深度阐明通用型细胞免疫治疗的作用机制，对新型通用型CAR-T不同层面的解析，促进新型免疫细胞治疗产品实现临床转化。
